# Chitosan Nanoencapsulated Exogenous Trypsin Biomimics Zymogen-Like Enzyme in Fish Gastrointestinal Tract

**DOI:** 10.1371/journal.pone.0074743

**Published:** 2013-09-10

**Authors:** Rakhi Kumari, Subodh Gupta, Arvind R. Singh, S. Ferosekhan, Dushyant C. Kothari, Asim Kumar Pal, Sanjay Balkrishna Jadhao

**Affiliations:** 1 Division of Fish Nutrition, Biochemistry and Physiology, Central Institute of Fisheries Education, Mumbai, Maharashtra, India; 2 Department of Physics and National Center for Nanomaterials and Nanotechnology, University of Mumbai, Mumbai, Maharashtra, India; University of California, Merced, United States of America

## Abstract

Exogenous proteolytic enzyme supplementation is required in certain disease conditions in humans and animals and due to compelling reasons on use of more plant protein ingredients and profitability in animal feed industry. However, limitations on their utility in diet are imposed by their pH specificity, thermolabile nature, inhibition due to a variety of factors and the possibility of intestinal damage. For enhancing the efficacy and safety of exogenous trypsin, an efficient chitosan (0.04%) nanoencapsulation-based controlled delivery system was developed. An experiment was conducted for 45 days to evaluate nanoencapsulated trypsin (0.01% and 0.02%) along with 0.02% bare trypsin and 0.4% chitosan nanoparticles against a control diet on productive efficiency (growth rate, feed conversion and protein efficiency ratio), organo-somatic indices, nutrient digestibility, tissue enzyme activities, hematic parameters and intestinal histology of the fish *Labeo rohita*. All the synthesized nanoparticles were of desired characteristics. Enhanced fish productive efficiency using nanoencapsulated trypsin over its bare form was noticed, which corresponded with enhanced (P<0.01) nutrient digestibility, activity of intestinal protease, liver and muscle tissue transaminases (alanine and aspartate) and dehydrogenases (lactate and malate), serum blood urea nitrogen and serum protein profile. Intestinal tissues of fish fed with 0.02% bare trypsin showed broadened, marked foamy cells with lipid vacuoles. However, villi were healthier in appearance with improved morphological features in fish fed with nanoencapsulated trypsin than with bare trypsin, and the villi were longer in fish fed with 0.01% nanoencapsulated trypsin than with 0.02% nanoencapsulated trypsin. The result of this premier experiment shows that nanoencapsulated trypsin mimics zymogen-like proteolytic activity via controlled release, and hence the use of 0.01% nanoencapsulated trypsin (in chitosan nanoparticles) over bare trypsin can be favored as a dietary supplement in animals and humans.

## Introduction

Advances in the understanding of gastrointestinal tract physiology have enabled the manipulation of digestibility and absorption of food constituents, chemicals and biomolecules to meet individual needs and goals in farm animal production. Exogenous enzyme supplementation is an important area of research in humans and animals. People with exocrine pancreatic insufficiency and cystic fibrosis frequently require supplemental pancreatic enzymes (which include proteolytic enzymes, lipases, and amylases). In addition, some people suffering from celiac disease [1] or Crohn’s disease [2] and perhaps from indigestion [3] may be deficient in pancreatic enzymes. Exogenous enzymes in animal feed are used for compelling reasons: shortage of preferred animal protein sources (fish meal), coupled with greater use of plant protein ingredients as well as the profitability in the fish, poultry and pig sectors, which are compete for feed resources among themselves and also with humans. Because global fish production is in decline, these industries will increasingly rely on protein sources of plant origin. In addition, the aquaculture sector, the fastest growing food sector [4], requires rapid growth of aquafeed production using cheaper plant-based ingredients to satisfy its demands. Exogenous enzymes enable them to digest plant-based ingredients containing more fiber, low quality protein and various anti-nutritional factors, which have been shown to lower nutrient digestibility [5]–[7]. Moreover, the metabolic activity during the growth phase across species including fish and shellfish (juvenile stage) is quite high, and consequently requires a nutrient-dense (mainly high protein) diet in order to optimise survival and growth. However, the digestive system is not developed enough to digest a nutrient-dense diet. Improper digestion and malabsorption of nutrients often have far-reaching effects that include reduced growth, impaired immunity, allergic reactions and poor wound healing. These problems can be rectified by incorporating exogenous enzymes and nutraceuticals in aquafeed. Enzymes manufactured by synthetic means or derived from plants, animals or microbial sources are increasingly being used as additives in animal feeds [8]–[9]. Supplementation of diet with pancreatic enzymes stimulated growth rate and protein utilization in *Salmo salar* [10] and with bovine trypsin significantly increased the proteolytic activity in common carp [11].

Although in theory exogenous proteolytic enzymes would not cause health problems, a serious condition called fibrosing colonopathy involving damage to the large intestines has resulted from the use of pancreatic enzymes in children with cystic fibrosis. In some cases, the problem was linked to the use of high supplemental amounts of enzymes [12]–[14]. However, the amount of enzymes used has not been linked to the problem in all reports [15]. In some cases, lower amounts of enteric-coated enzymes have caused fibrosing colonopathy [16]. It is believed to be the result of unknown interactions between the enteric coating and the enzymes themselves causing damage to the intestines of children with cystic fibrosis [17]. In animals, damage to mucosa can increase the chance of infection. In animals, studies discussing safety of proteolytic enzymes are scarce.

Applications of exogenous enzymes in food/feed have certain limitations due to their thermolabile nature, pH specificity, heavy metal salt, various inhibitors etc. Additionally, use of unregulated proteolytic enzymes may cause damage to intestines and cause stress. To overcome these problems, an efficient delivery system has to be developed to maximize the efficiency of exogenous enzymes and enhance safety. A controlled release system for robust proteolytic enzyme (like monoproteases) is an option which has not been tried so far. A number of polymeric compounds and lipids are being used in medical fields to improve the delivery efficiency of enzymes, drugs, hormones, vaccines (DNA and RNA) or genes. Among those investigated, chitosan acts as an excellent carrier system for nutraceuticals and other biomolecules because of its versatile properties including biocompatibility, biodegradability, membrane forming ability, reactive surface functional groups for easy surface modification, low toxicity, immunomodulation, membrane adhesiveness, improved stability and enhanced permeability of epithelial membrane [18]–[19]. Chitosan is a base material for immobilization of several enzymes, as it exhibits increased thermostability compared to the free enzymes [19] and may help in protecting the biomolecules from the adverse effect of temperature, pH and endogenous enzyme activity [20].

Nanotechnology is ushering a new era in the field of medicine and the agri-food and feed sectors. Nanoscale drug systems have the advantage that they can circumvent rapid recognition by the immune system and deliver drugs to cells with high efficiency compared with microparticle based systems [21]. Nanoparticles have been studied as carriers for oral drug delivery to improve drug stability and bioavailability across the GI mucosa [22]. A number of polymeric nanoparticles have been synthesized and studied in the past few years as promising drug delivery systems which improve delivery efficiency and reduce side-effects of drug toxicity [23]–[24] including chitosan nanoparticles for controlled delivery of vitamin C in fish [25].

Fish are becoming popular as a research model of human pathologies because of their easy availability, high fecundity, rapid breeding and ease of maintaining and their high genetic and organ system homology to humans. One of the areas where fish models have contributed enormously to the understanding of human pathologies is the area of gastrointestinal diseases and microbe-host interactions [26]. The zebrafish model of inflammatory bowel disease [27]–[29] and glafenine-induced intestinal injury amelioration are effective as the gastrointestinal system of fishes, like humans, possess liver, pancreas, gall bladder and a linearly segmented intestinal track with absorptive and secretory functions [30]–[31] with proximal-distal functional specification and containing absorptive enterocytes having apical microvilli, an intestinal brush border [32] and other structures, goblet cells and enteroendocrine cells [30]–[32]. Information generated in fish research also has important applications for aquaculture and the fishery industry itself which contributes to food and nutritional security.

This premier report on fish model, with implications for animal species and humans, discusses the results of the hypothesis that nanoencapsulation of trypsin with chitosan releases enzyme in controlled manner and biomimics zymogen-like activity, thus improving safety of use in addition to production efficiency in fish.

## Materials and Methods

### Chemicals

Chitosan with a deacetylation degree of 90% was purchased from Sigma-Aldrich (St. Louis, MO). All other chemicals were of analytical and reagent grade.

### Purification of chitosan

One gram of chitosan was dissolved in 15 mL of 1 M sodium hydroxide (NaOH) solution by heating at 50°C with vigorous stirring for 2 hours. The chitosan solution was filtered using a Nalgene filter (Nalgene, Rochester, NY) followed by washing with deionized water. The filtered chitosan was dried overnight at 40°C and then dissolved in 0.1 M acetic acid. Using a Buchner filter unit (Fisher Scientific, Houston, TX) insoluble residues were removed from the chitosan solution. The pH of the solution was adjusted to 7.0 using 1 M NaOH, which resulted in purified chitosan precipitate. The precipitated chitosan was collected by centrifugation and washed with deionized water. The chitosan was freeze-dried in lyophilizer (Labconco, Mo). This lyophilized chitosan was further used for nanoparticle preparation [33]–[34].

### Synthesis of chitosan nanoencapsulated trypsin nanoparticles

Chitosan nanoparticles (NPs) were prepared according to the ionotropic gelation procedure [35] with slight modification. Chitosan (0.4 g) flakes were dissolved in 10 ml glacial acetic acid and the pH was adjusted to 7.0 by addition of sodium hydroxide. This solution was vigorously stirred with a magnetic stirrer at ambient temperature until the solution became clear. The total volume was made up to 100 ml. It was then sterile-filtered using a 0.22 µm syringe filter. Then tripolyphosphate (TPP) (1 mg/ml) solution was added drop-wise to the chitosan solution while stirring. Nanoparticles were formed immediately upon mixing of TPP and chitosan solutions as molecular linkages were formed between TPP phosphate and chitosan amino groups. The solution was stored at 4°C for further use. Enzyme solution was prepared by dissolving enzyme trypsin (10 mg/ml) in 67 mM phosphate buffer at pH 7.6. Trypsin was encapsulated in chitosan NPs by drop-wise addition of enzyme into the chitosan-TPP solution and homogenized for 5 min at 4°C. These nanoparticles were used for preparation of experimental diets. Enzyme encapsulation efficiency of nanoparticles [36] was determined by the separation of nanoparticles from the aqueous medium containing non-associated enzyme by centrifugation at 15000 rpm at 4°C for 30 min. The amount of free enzyme in the supernatant was measured by UV spectrophotometry at 280 nm using the supernatant of the non-encapsulated enzyme nanoparticles as basic correction. Trypsin encapsulation efficiency percentage (EE %) was calculated using the following formula: 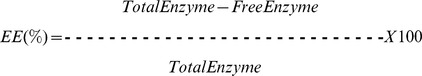



### Characterization of chitosan and encapsulated trypsin nanoparticles

Mean particle size, poly-dispersity index (size distribution) and zeta-potential of chitosan-encapsulated trypsin nanoparticles were determined by Delsa™ Nano-C (Brea, CA) at room temperature in the triplicate. The morphological characteristics of the chitosan-encapsulated trypsin nanoparticles were examined by using a tapping-mode atomic force microscope (AFM) (JPK NanoWizard® AFM, Berlin, Germany). One drop of chitosan solution was placed onto a freshly cleaved piece of mica and air-dried.

### Ethics Statement

The research undertaken complies with the current animal welfare laws in India. The study was approved by the Board of Studies and authorities of the Central Institute of Fisheries Education (Deemed University), Mumbai-61. The care and treatment of animals used in this study were in accordance with the guidelines of the CPCSEA [(Committee for the Purpose of Control and Supervision of Experiments on Animals), Ministry of Environment & Forests (Animal Welfare Division), Govt of India] on care and use of animals in scientific research. As the experimental fish i.e. *L. rohita* is a commercially important and not endangered fish, the provisions of the Govt of India’s Wildlife Protection Act of 1972 are not applicable for experiments on this fish.

### Fish procurement, rearing and experimental conditions

Fingerlings of *Labeo rohita* were brought from Aarey fish farm (Goregaon, MS, India) and acclimated in a circular tank with a capacity of 2 tonnes for a period of 15 days and fed a diet of 30% crude protein. The experiment was conducted for a period 45 days. At the beginning of the study, *L. rohita* fingerlings were weighed individually and fifteen fishes (mean wt 2.8 g) were stocked in each of fifteen plastic tubs. Two hundred twenty five *L. rohita* fingerlings were randomly distributed into five experimental groups with three replicates each, following a completely randomised design. Round the clock aeration was provided to all the tubs and manual water exchange was done every other day. Water temperature, pH, dissolved oxygen, nitrite-N, nitrate-N and phosphate were analyzed every week using standard methods. Water quality parameters recorded during the study [37] were as follows: dissolved oxygen between 5.8–7.3 mg/L, and temperature range of 23.6–27.5°C (dissolved oxygen and temperature meter, Merck, Germany); pH 7.5–8.4 (digital pH meter, LABINDIA, Mumbai); negligible free carbon dioxide (titrimetric method); total hardness228–245 mg L^− 1^ (carbonate hardness test kit, Merck, Germany); ammonia 0.16–0.25 (at 635 nm by phenate method); nitrite 0.002–0.004 and nitrate 0.02–0.06 (543 nm wavelength).

### Experimental diets and feeding

Five experimental groups were fed one of the following diets: a control (basal diet), or a basal diet supplemented with 0.4% chitosan NPs; 0.02% bare trypsin and 0.01% trypsin encapsulated with chitosan (0.4%) NPs; or 0.02% trypsin encapsulated with chitosan (0.4%) NPs. The composition of the basal diet by percentage was as follows: soy bean meal, 39.5; fish meal, 5; wheat flour, 12; mustard oilcake meal, 21; rice polish 14.5; oil mix, 6; carboxy methyl cellulose, 1; vitamin and mineral mixture 0.5; and chromic oxide, 0.5. The mean proximate (n = 3) composition (% DM basis): crude protein 28.49±0.29, ether extract 9.13±0.07, ash 8.55±0.11, total carbohydrate 53.81±0.40 and digestible energy 411±0.5 kcal. All the diets were isonitrogenous and isocaloric and were supplemented with butylated hydroxytoluene (0.02%).

All the ingredients were weighed properly as per requirements and mixed properly in a plastic container to form dough with the addition of the necessary quantity of water. When the dough was formed, the calculated quantity of the oils were added to it and mixed well. The dough was then transferred to an aluminum container, which was then placed in a pressure cooker for steaming/cooking. The steaming was done for 30 min. The pressure cooker was then removed from the flame and set aside to cool. The steamed dough was then taken out and was cooled further. To prevent their loss, vitamins and minerals along with the enzymes were added when the steamed dough was completely cooled. After incorporation of these elements, the dough was mixed properly and was pressed through a hand pelletizer to get uniform sized pellets, which were spread on a sheet of paper and were initially air dried. After that the feed was transferred to trays and was kept in a hot air oven overnight for complete drying at 35°C – 40°C. After drying, the pellets were packed in polythene bags and were sealed airtight and labeled according to the treatments. Feeding was carried out twice daily (9:00 a.m. and 6:00 p.m.) at 5% of body weight initially, and subsequently adjusted based on daily intake. Fish in each tub were weighed every 15 days to assess their growth.

### Digestibility and sampling

Apparent digestibility coefficients (ADCs) were measured by the indicator method using 0.5% chromium oxide as a marker [38] during the last 20 days of study. Following the initial five-day acclimation to the diets; faecal materials were collected daily from the 30th to 45th day, for determining the digestibility of nutrients. Faecal material was collected manually by siphoning and straining through a fine-meshed net. Three hours after the first (9:00 a.m) feeding, uneaten feed, together with the faeces, was siphoned out and discarded. At 2:00 p.m., faecal material was collected and dried to constant weight at 60°C. Wet ashing of diets and faecal matters was carried out according to AOAC [39], and the chromium content of feed and faecal matters was determined with a flame ionization atomic absorption spectrophotometer (AAS 4129, Electronics Corporation of India, Hyderabad). The ADC of dry matter was calculated as: 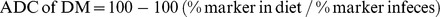
While ADC of a nutrient was calculated as: 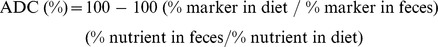



### Chemical analysis of experimental diets and faecal samples

The proximate composition of all the diets was determined by the standard methods of the Association of Official Analytical Chemists [39]. In brief, moisture content was determined by drying at 105°C to a constant weight, nitrogen content was estimated by Kjeltec (2200 Kjeltec Auto distillation, Foss Tecator, Sweden), crude protein (CP) was estimated by multiplying nitrogen percentage by 6.25, ether extract (EE) was measured with a Soxtec system (1045 Soxtec extraction unit, Tecator, Sweden) using diethyl ether (boiling point 40–60°C) as a solvent, and ash content was determined by incinerating samples in a muffle furnace at 600°C for 6 h. Total carbohydrate (CHO) was calculated by difference: 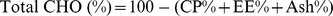



### Growth study

Fish in each tub were bulk-weighed every 15 days to assess their growth. Growth performance of fingerlings was evaluated in terms of specific growth rate (SGR), protein efficiency ratio (PER) and food conversion ratio (FCR) based on the following standard formulae.
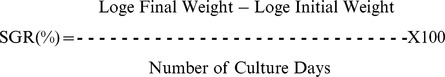


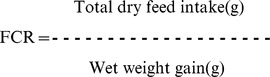


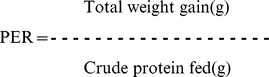



### Blood collection

At the end of the feeding trial, a few fishes from each treatment group were anaesthetized with clove oil (50µl L^−1^) until they become motionless. Blood was collected from the caudal vein using a syringe, and then transferred immediately to the test tube containing a pinch of EDTA powder (as anticoagulant) and shaken gently in order to prevent haemolysis. For serum, blood was collected from the same treatment groups and transferred to the new test tube, allowed to clot for 2 hours, centrifuged (3000 x *g* for 5 min) and then kept at –80 °C until use.

### Organo-Somatic Indices

After the clove oil anaesthetic treatment, the fish were removed from water when they were unresponsive to external stimuli. Liver and viscera were dissected out and immediately placed on ice. They were weighed separately with accuracy up to 1 mg for estimation of somatic indices. Hepato-somatic index and viscero-somatic index were determined as a proportion of weight of organ/tissue to the total body weight multiplied by 100.

### Blood glucose and blood urea nitrogen (BUN)

Blood (500µl) was de-proteinized by mixing with 4.75 ml zinc sulphate followed by addition of 4.75 ml barium hydroxide. The solution was mixed vigorously and filtered and the supernatant was used for colorimetric estimation of glucose by the Nelson-Somogyi method at 540 nm against a blank. The BUN was determined colorimetrically by the diacetyl monoxime method at 525 nm.

### Serum total protein, albumin, and globulin and A: G ratio

Total serum protein (by the biuret method) and albumin (by the bromocresol green binding method) were determined colorimetrically at 630 nm using the diagnostic kits (Qualigens Diagnostics, Mumbai, India). Globulin (G) was calculated by subtracting albumin (A) values from total serum protein and the A: G ratio was subsequently calculated. The detailed methodology followed for these estimations have been described elsewhere [5].

### Digestive and metabolic enzyme analysis

The liver, muscle and intestine of the fish were removed carefully and were weighed as mentioned above. A 5% homogenate was prepared with chilled sucrose solution (0.25 M) in a glass tube using a Teflon-coated mechanical tissue homogenizer. The homogenate was centrifuged at 5000 rpm for 10 minutes at 4°C in a cooled centrifuge machine. The supernatant was stored at 4°C for further analysis.

Protease activity was determined as described by [40]. The reaction mixture consisted of 1% casein in 0.05 M Tris-PO4 buffer (pH 7.8) and was incubated for 5 min at 37°C. The tissue homogenate was then added. Ten minutes later the reaction was stopped by the addition of 10% TCA, followed by filtration of the samples. The reagent blank was made by the addition of tissue homogenate just before stopping the reaction without incubation. One unit of enzyme activity was defined as the amount of enzyme needed to release acid-soluble fragments equivalent to 0.001 A280 per minute at 37°C and pH 7.8.

Standard methods that were followed in our laboratory for biochemical analysis of lactate dehydrogenase (LDH) (E.C. 1.1.1.27), malate dehydrogenase (MDH) (E.C. 1.1.1.37), aspartate amino transferase (AST) (E.C.2.6.1.1) and alanine amino transferase (ALT) (E.C.2.6.1.2), and protein concentration have been recently published [41].

### Preparation of tissue samples and histology of intestine

The histological study of intestinal tissue was conducted by sacrificing the fish from each experimental group at the end of the experiment. The fish were sacrificed and tissues were collected as mentioned above. Intestine of fish from different experimental groups were taken and fixed separately in 10% buffered formalin and were dehydrated in 90% alcohol for one hour, and three times in absolute alcohol for 45 minutes each. The samples were then cleared two more times in xylene for 30 minutes and embedded in paraffin for 45 minutes three times. The samples were then blocked and were allowed to cool. They were then cut on a rotator microtome at 7 μm and the mounted sections were dewaxed and dehydrated serially in alcohol. Then the slides were washed in tap water for 1 minute, stained in haematoxylene for 12 minutes, washed in tap water, dipped in 2% acid alcohol and washed in tap water, followed by Scott’s tap water substitute. The sections were dehydrated with 50%, 70% and 90% alcohol for 1 minute. Finally the stained sections were cleared in xylene for 5 minutes and mounted with DPX. Prepared slides were examined and photographed under a light microscope.

### Statistical analysis

Mean values of all parameters were subjected to one-way ANOVA to study the treatment effect, and Duncan's multiple range tests were used to determine the significant differences between any two means, if they were significant. Comparisons were made at 5% probability level. All the data were analyzed using statistical package SPSS (Version 19).

## Results

### Synthesized nanoencapsulated trypsin has desired characteristics

**Figure 1:**
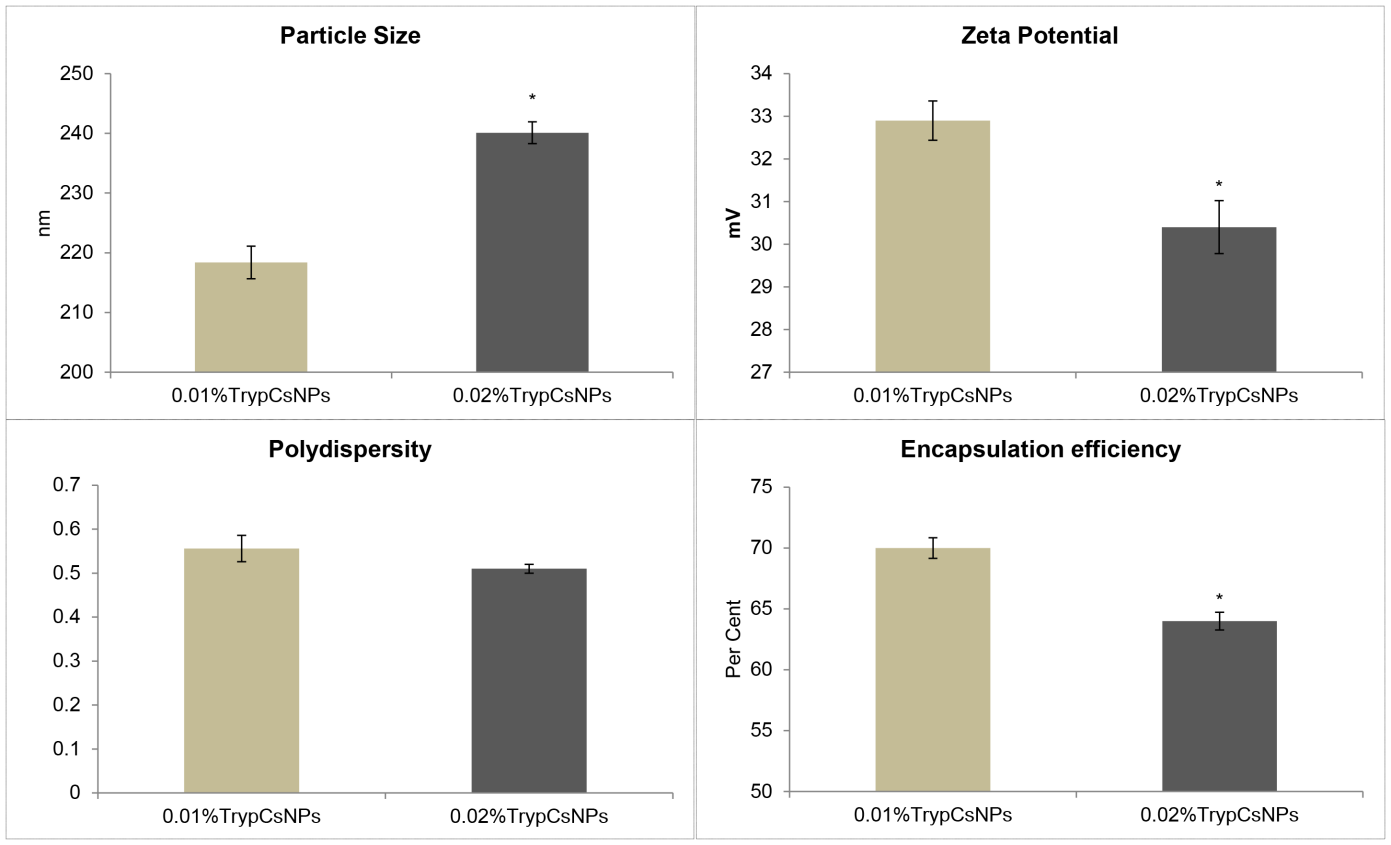
Physico-chemical characteristics of 0.01% and 0.02% trypsin encapsulated in 0.4% chitosan nanoparticles (TrypCsNPs). Means bearing different superscript letters differ significantly (*P<0.05 by student t test).Values represent means ± standard error of triplicate observations.

**Figure 2:**
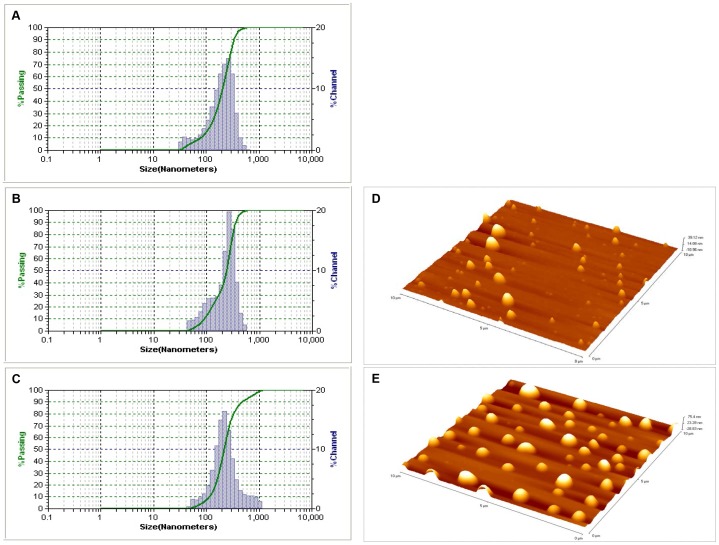
Particle size distribution A] 0.4% chitosan nanoparticles B] 0.01% trypsin encapsulated in 0.4% chitosan nanoparticles, C] 0.02% trypsin encapsulated in 0.4% chitosan nanoparticles **and three-dimensional images** D] 0.01% trypsin encapsulated in 0.4% chitosan nanoparticles, E] 0.02% trypsin encapsulated in 0.4% chitosan nanoparticles.

The 0.4% chitosan nanoparticles had a mean (n = 3) particle size of 147±2.25 nm, zeta potential of 37.1±0.54 mV and poly dispersity index of 0.667±0.01. Enzyme nanoparticles employing two different levels of trypsin (0.1% and 0.02% trypsin) using 0.4% chitosan as a vehicle were prepared. The physical and chemical characteristics of nanoencapsulated trypsin are given in Fig 1 and particle size distributions are given in Fig 2 (A to C). Comparison of 0.01% and 0.02% nanoencapsulated trypsin indicated that increasing the percentage of trypsin increased (P<0.05) the particle size (by 22 nm), and lowered the zeta potential (P<0.05) and encapsulation efficiency (P<0.05) (by 6%). Three-dimensional (3-D) imaging and analysis by atomic force microscope (AFM) of the two chitosan-nanoencapsulated trypsins revealed that the nanoparticles exhibited spherical shape with a homogenous distribution (Figure 2D and E).

### Nanoencapsulated trypsin enhanced fish performance over its bare form

**Figure 3:**
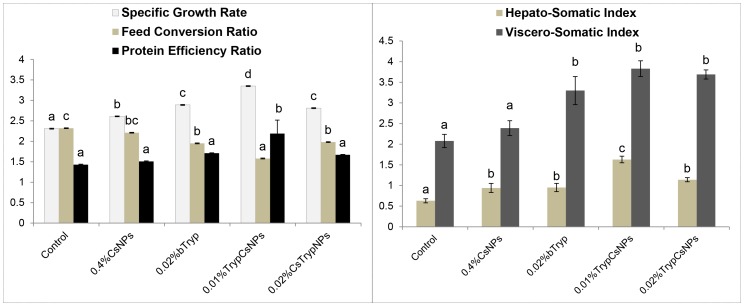
Productive performance and hepatosomatic- and viscero-somatic index of fish on diets containing 0.4% chitosan nanoparticles (CsNPs), 0.02% bare trypsin (bTryp), and 0.01% or 0.02% trypsin encapsulated in 0.4% chitosan nanoparticles (TrypCsNPs). Means bearing different superscript letters differ significantly (P<0.05). Values represent means ± standard error of triplicate observations.

The data on growth and performance (Fig 3) indicated that feeding bare trypsin increased specific growth rate (SGR) and improved feed conversion ratio (FCR). Nanoencapsulation of trypsin at low doses further potentiated its nutritional benefits with regard to productive performance. Even at half the dose level of bare trypsin, in nanoencapsulated form, the productive efficiency in terms of SGR, FCR and PER was significantly (P<0.05) higher than with bare trypsin. Moreover, nanoencapsulation of trypsin at the same dose as bare trypsin (i.e. 0.02%) offered no additional production benefits as these attributes were similar in bare and nanoencapsulated 0.02% trypsin. Hepatosomatic index (HSI) and viscero-somatic index (VSI) data (Fig 3) indicated that chitosan NPs, bare and nanoencapsulated trypsin significantly increased HSI. The 0.01% nanoencapsulated trypsin had significantly highest HSI among all the groups. Trypsin, in either form, increased VSI significantly (P<0.05).

### Nanoencapsulated trypsin enhanced nutrient digestibility in fish over its bare form

**Figure 4:**
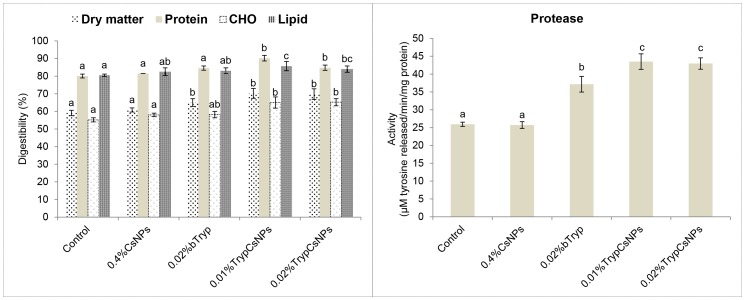
Digestibility of diets and intestinal protease activity in fish fed these diets containing 0.4% chitosan nanoparticles (CsNPs), 0.02% bare trypsin (bTryp), and 0.01% or 0.02% trypsin encapsulated in 0.4% chitosan nanoparticles (TrypCsNPs). Means bearing different superscript letters differ significantly (P<0.05). Values represent means ± standard error of triplicate observations.

Feeding chitosan NPs had no effect on digestibility of dry matter and other nutrients. Feeding trypsin in either form increased the digestibility of dry matter (Figure 4). The apparent digestibility of protein was significantly increased with nanoencapsulated trypsin supplementation. While digestibility of carbohydrate in the nanoencapsulated trypsin group was significantly higher than the control and chitosan NPs-fed groups, it was not different from bare trypsin-fed group. The digestibility of lipid in the nanoencapsulated 0.01% trypsin group was significantly higher than in control, chitosan NPs and bare trypsin groups, and that of nanoencapsulated 0.02% trypsin was higher than control but similar to the other groups. Protein digestibility corresponded with intestinal protease activity (Fig 4). Feeding chitosan NPs had no effect on protease activity. However, feeding the nanoencapsulated form of trypsin at both half the dose rate and at the same dose rate of bare trypsin resulted in a significant increase (P<0.05) of intestinal protease activity.

### Enhanced transaminases and reduced dehydrogenases with nanoencapsulated trypsin over its bare form

**Figure 5:**
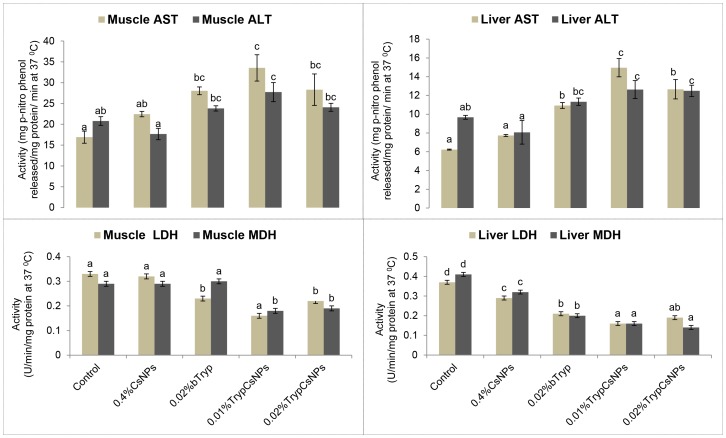
Activities of aminotransferases and dehydrogenases in fish fed diets containing 0.4% chitosan nanoparticles (CsNPs), 0.02% bare trypsin (bTryp), and 0.01% or 0.02% trypsin encapsulated in 0.4% chitosan nanoparticles (TrypCsNPs). Abbreviations: Aspartate aminotransferase (AST), alanine aminotransferase (ALT), lactate dehydrogenase (LDH) and malate dehydrogenase (MDH). Means bearing different superscript letters differ significantly (P<0.05). Values represent means ± standard error of six observations.

Activity of the liver and muscle transaminases and the dehydrogenases of *L. rohita* fingerlings in the different experimental groups are presented in Fig 5. Fish fed nanoencapsulated trypsin at both 0.01 and 0.02% levels had higher liver ALT and the 0.01% dose showed higher AST activity than bare trypsin. Muscle ALT, but not muscle AST, in fish fed nanoencapsulated trypsin at both the 0.01 and 0.02% level was higher than in fish fed bare trypsin. Compared to control, LDH and MDH activity in the liver was reduced in groups fed chitosan or trypsin in either form. While trypsin in either form reduced muscle LDH, only trypsin in nanoencapsulated form reduced both muscle LDH and MDH.

### Enhanced plasma components and serum protein profile with nanoencapsulated trypsin over its bare form

**Figure 6:**
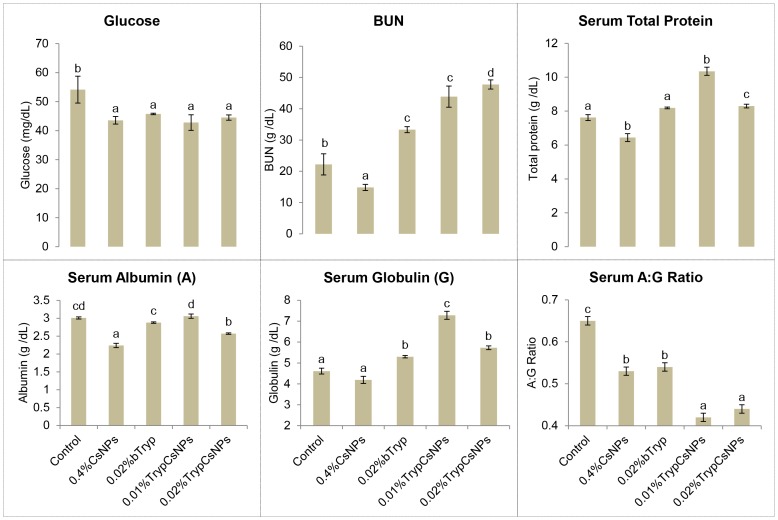
Plasma components and serum protein profile in fish fed diets containing 0.4% chitosan nanoparticles (CsNPs), 0.02% bare trypsin (bTryp), and 0.01% or 0.02% trypsin encapsulated in 0.4% chitosan nanoparticles (TrypCsNPs). Means bearing different superscript letters differ significantly (P<0.05). Values represent means ± standard error of six observations.

The plasma components and serum protein levels of the different experimental groups are given in Fig 6. Compared to control, blood glucose was decreased in all of the treatment groups. While BUN was decreased in chitosan NPs-fed group, BUN was increased in both trypsin-fed groups. Significantly higher total serum protein level was found in chitosan nanoencapsulated trypsin groups. Trypsin in bare as well as nanoencapsulated form increased serum globulin. Chitosan NPs and trypsin in either form decreased serum A: G ratio.

### Histological study of intestine indicates safety of nanoencapsulated trypsin over its bare form

**Figure 7:**
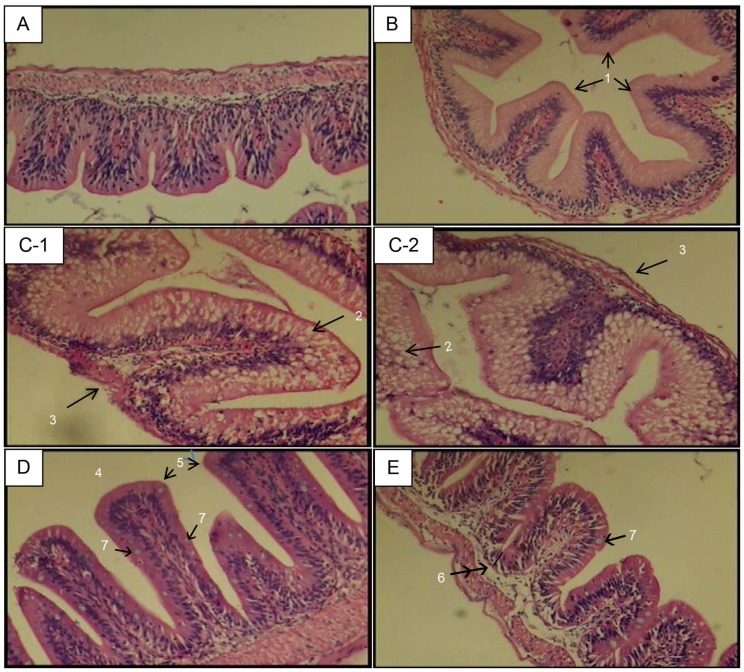
Intestine histology of fish fed control, 0.4% chitosan nanoparticles, 0.02% bare trypsin and and 0.01% or 0.02% nanoencapsulated trypsin containing diets (H&E, 40X). In control group (A), intestinal mucosa is lined by regularly-packed villi with continuous basement membrane. In 0.4% chitosan nanoparticles fed fish (B), mildly swollen apical surface of villi (1) are noticed. In 0.02% bare trypsin (C-1 and C2), broadened villi, marked foamy cells with lipid vacuoles (2), atrophied submucosal layer and muscularis (3) were evident. In 0.01% trypsin encapsulated in 0.4% chitosan nanoparticles (D), longer villi (4) with healthy apical surface (5) and improved morphological features besides continuous basement membrane are evident. Crypt depth was less in treated groups (6). Distinct mucin producing goblet cells (7) distributed along the villi in nanoencapsulated trypsin groups indicates better gastro-intestinal health. Villi in 0.02% trypsin encapsulated in 0.4% chitosan nanoparticles (E) resembled those in control group (A). Effectiveness and safety of dietary nanoencapsulated trypsin at half the dose rate (0.01%) (D) of bare trypsin (0.02%) (C-1 and C-2) is evident from healthier villi with more height and absorptive surface.

Intestine histology of *Labeo rohita* fingerlings fed the control diet showed intact architecture (Fig 7A), and histology of 0.4% chitosan NPs-fed fish did not show much difference from control (Fig 7B). Intestinal tissues of fish fed with the 0.02% bare trypsin supplemented diet showed broadened villi, marked foamy cells with lipid vacuoles, atrophied submucosal layer and muscularis (Fig 7C-1 and 7C-2). In general, villi were healthier in appearance and had improved morphological features after being fed chitosan nanoencapsulated trypsin compared to bare trypsin. The villi were longer in fish fed with 0.01% chitosan nanoencapsulated trypsin (Fig 7D) than 0.02% chitosan nanoencapsulated trypsin, which slightly resembled the control group (Fig 7E).

## Discussion

Nanoparticles have been studied as carriers for oral drug delivery to improve drug stability and bioavailability across the GI mucosa [22]. However, there are no reports on controlled release systems for proteolytic enzymes with potential use in the biomedical and animal feed industries. The present study clearly shows benefits for productive performance and the healthiness of intestinal villi from chitosan-encapsulated trypsin more so than from bare trypsin. Such a positive impact on enzyme stability and its bioavailability was expected from nanoencapsulation prepared by ionic gelation method. Ionotropic gelation between chitosan and tripolyphosphate leads to the formation of nano-sized colloidal systems. The colloidal particles have electrostatic interactions with charged molecules under aqueous physiological conditions [19] and incorporated protein, in this case trypsin, is protected against degradation in *in vivo* applications [34], [42].

Nanoencapsulated trypsin had satisfactory characteristics. Chun et al. [43] reported that chitosan nanoparticles sized between 35–190 nm and having zeta potential 35–42 mv are promising carriers for delivery of proteins. The zeta potential figures of nanoparticles were indicative of good particle stability. Increasing trypsin concentration from 0.01% to 0.02% decreased the encapsulation efficiency of chitosan nanoparticles, indicating that 0.4% chitosan NPs can hold more than 0.01% but less than 0.02% trypsin.

Increased specific growth rate, improved feed conversion and protein efficiency ratio in the experimental animals suggest that even after processing during feed manufacture the chitosan nanoencapsulated trypsin remained stable and active within the gut of the fish. The observations corroborate with Gole et al. [44], who reported that pepsin immobilized on a gold nanoparticle surface was more stable when compared with the free enzyme. Increased growth was shown in shrimp *Penaeus japonicas* [45] which were supplemented with amylase in a freeze-dried nylon-protein microcapsule in order to circumvent potential inactivation.

The apparent nutrient digestibility values corresponded with the growth performance of fish fingerlings in this study. While studies with enzyme mixtures are abundant, and though limited studies with mono-protease supplementation [46] also exist, there are no *in vivo* studies of mono-component proteolytic enzymes, including encapsulated enzymes, depicting the overall health of the intestine through histological investigation. Gut enzyme profile is the indicator of nutrient digestibility and utilization. The significantly higher activity of protease in fish fed nanoencapsulated trypsin (0.01% and 0.02%) clearly indicates a correlation between formulated diet intake and digestive enzyme activity, resulting in diet-related growth differences. Similar diet-related differences in growth and digestive enzyme activity have been reported with rainbow trout [47]. Common carp fed a diet supplemented with bovine trypsin showed increased proteolytic activity, and the increase in the activity was correlated with the bovine trypsin proportion [46]. In studies by Dalsgaard et al. [48], supplementing with glucanase or protease in a salmonid diet containing soybean meal significantly improved the apparent digestibility of all dietary nutrients. In another study [49], a commercial protease supplementation of a diet containing a mixture of rapeseed and pea was found to increase protein, lipid, energy and dry matter digestibility. Thus, the influence on digestibility of protein, fat and lipids in response to dietary proteolytic enzyme supplementation appears to depend on composition of the diet. In chickens, mono-component protease supplementation improved feed to gain as well as the digestibility of fat and CP, regardless of dietary protein or energy concentration [50]. Unaffected protease activity in the chitosan-fed group agrees with Han et al. [51]. However, the increased specific growth rate and feed conversion ratio in fish may be result of its antibacterial activity, and the findings corroborate with the reported results [52] in Tilapia.

The chitosan NPs and nanoencapsulated trypsin significantly increased hepatosomatic index (HSI) compared with control. The 0.01% nanoencapsulated trypsin had the significantly highest HSI. Trypsin, in either form, significantly (P<0.05) increased viscero-somatic index (VSI). The HSI and VSI was decreased in fish supplemented with a 0.1% and 0.15% commercial enzyme complex (neutral protease, b-glucanase and xylanase) during a 12-week study [53]. It is to be noted that the enzyme concentrations used in the feed in this study were 5 to 15 times lower than used by Lin et al. [53].

The major source of energy in teleosts is amino acids [54]. Provision of more gluconeogenic substrates through increased ALT and AST [55] indicates increased demand for energy, which occurs under conditions of increased growth or stress. In the present study, the activities of amino acid metabolism enzymes AST and ALT in muscle and especially liver were increased in chitosan NPs, trypsin- and nanoencapsulated trypsin-fed fish, which indicated increased utilization of dietary protein corresponding with an increase in growth (protein synthesis) in respective groups. In a 60-day feeding trial investigating the effects of dietary rapeseed meal levels on feed intake, growth, digestion and protein metabolism in juvenile cobia, Luo et al. [56] has shown significantly decreased activity of liver ALT and AST with decreased growth rate. Aminotransferases catabolize amino acids and transfer amino groups to alpha-keto acids. This can be attributed to high protein digestibility in these groups and controlled breakdown and release of amino acids in encapsulated trypsin-fed fish, creating a surplus pool of the available essential amino acids, and so the keto acids may be oxidised, thereby increasing the activities of ALT and AST [56]–[57]. There is a negative correlation between growth rates and LDH and MDH activity levels and a positive correlation between AST and growth [58]–[59]. This relationship was noticed in this study not only for transaminases but also for dehydrogenases (LDH and MDH), especially in nanoencapsulated trypsin-fed groups where growth was significantly more pronounced than in others.

Blood glucose was decreased in all the treatments. Change in the glucose levels is a secondary stress response providing required energy to cope with stressful conditions [60]. Hyperglycemia is due to stimulation of glycolysis and gluconeogenesis from protein and lipid sources [60]. Changes in serum blood urea nitrogen (BUN) concentration are indicative of the whole body status of amino acid metabolism and utilization in animals [61]. While BUN was decreased in the chitosan NPs-fed group, BUN was increased in either form of trypsin-fed groups. In early weaned piglets BUN was found to be reduced (P < 0.05) in response to dietary supplementation of chitosan and galactomannan-oligosaccharides during a two week study [62]. Similarly, Jo et al. [63] reported increased (P<0.05) BUN in growing pigs fed diets with protease-containing exogenous enzymes. Increased serum BUN suggests a potential breakdown of protein in animals due to trypsin supplementation after satisfying nutritional requirements.

An increase in the value of serum total protein, albumin and globulin levels is an indication of better immunity in animals [64]. Significantly higher total serum protein levels were found in chitosan nanoencapsulated trypsin groups. Trypsin feeding in its bare form as well as in the nanoencapsulated forms increased serum globulin. Serum A: G ratio indicated that chitosan NPs and trypsin in bare form and encapsulated forms improved serum A: G ratio and thus immunity. Dietary chitosan has been reported [65] to enhance the immunological properties of *C. carpio*. Similarly, serum total protein concentration was increased (P<0.05) in response to dietary supplementation of chitosan and galactomannan-oligosaccharids in early weaned piglets during a two week study [62]. In another study [66], 0.25 to 0.5% dietary supplementation of chitosan enhanced hematological profiles (lymphocyte count and total WBC) and 0.25% chitosan reduced (P<0.05) experimental mortality from environment-induced stresses (low oxygen, salinity and temperature) in rainbow trout. Abd El-Latif et al. [67] reported serum total protein and globulin in chickens fed a commercial multienzyme product.

Good intestinal mucosal morphology is the foundation of nutrient absorption and animal growth. Histologically, the intestine was not significantly altered by supplementation with 0.4% chitosan except for slightly swollen villi. In another study, statistically nonsignificant change from smooth in control to rough apical surface of intestinal villi and increased growth of broiler chickens fed 4 g/kg dietary chitosan was reported [68]. The 0.02% bare trypsin-fed group showed dilated swollen mucosal epithelium (villous broadening), marked foamy cells with lipid vacuoles, reduced crypt depth, and atrophied submucosal layer and muscularis. Compared to 0.02% bare trypsin fed group, in the nanoencapsulated trypsin group villi were longer in height with more surface area, showed more architectural symmetry and had a healthy appearance with distinct goblet cells distributed along the villi indicating better gastro-intestinal health. These cells secrete mucin, which destroys pathogens [69], into the mucus layer and also help maintain a suitable thickness of this layer in the intestine despite normal slough off. A greater number of goblet cells per villus have been reported to correlate with improved feed conversion ratio, growth performance and intestinal proteolytic enzyme activity in rainbow trout fed different nutritional supplements (yeast, S. cerevisiae and seaweeds) [70]–[71]. The villi were longer with more improved morphological features in the 0.01% than in the 0.02% nanoencapsulated trypsin group, which slightly resembled control. Crypt depth was less in treated groups. Increased villus length and reduced crypt depth were observed in the germfree animals [72]. The crypt epithelium primarily functions in epithelial cell renewal/regeneration [73]. This improvement of small intestine morphology corresponded with increased nutrient absorption in the gut and growth performance of fish. Chitosan nanoencapsulated trypsin was safe to feed, as indicated by protected normal mucosal epithelium. Increasing concentration from 0.01% to 0.02 had no effect on these parameters of intestinal morphology. These results are consistent with that of Iji et al. [74] who showed that addition of a high dose of enzyme to wheat-based diets had no effect on villus height, crypt depth or villus surface area of intestine of broilers. Reduction of villus height and surface area can reduce the absorption of nutrients. Therefore, the improved performance observed in fish fed with 0.01% nanoencapsulated trypsin may also be correlated with the morphology (increased absorptiveness) of small intestine [75] along with the release of simple sugars and protein.

The preceding discussion of significant positive effects on the performance of animals along with the safety of chitosan nanoencapsulated functional trypsin in the present experiment supports the reasons why chitosan is the most valued and preferred vehicle for preparing compounds intended for controlled release in the intestine through oral delivery in so many investigations. This delivery system has been successfully employed for the controlled oral delivery of proteins like insulin [76], drugs like the antitumor compound Gemcitabine [77] and other compounds of nutritional significance such as vitamin C [25] and yeast RNA/nucleotide [our unpublished data] with more pronounced desired effects. Recently, we reported pronounced *in vivo* biological effects due to the controlled release of a hormonal protein preparation encapsulated in chitosan nanoparticles compared to bare hormone [78]. Controlled delivery using chitosan nanoparticles also allows the preparation of organic solvent-free mucoadhesive particles [79].

In conclusion, enhanced performance and intestinal histological safety in the studied fish indicates that trypsin nanoencapsulated with chitosan mimics zymogen-like proteolytic activity, and the use of 0.01% nanoencapsulated trypsin over bare trypsin can be favored as a dietary supplement in animals and humans. Controlled release of enzyme in the study also indicates that chitosan nanocarriers are good vehicles for delivery of proteolytic enzymes or proteins where slow release is desired.
